# High sex ratios in rural China: declining well-being with age in never-married men

**DOI:** 10.1098/rstb.2016.0324

**Published:** 2017-07-31

**Authors:** Xudong Zhou, Therese Hesketh

**Affiliations:** 1 Institute for Social and Family Medicine, Zhejiang University, 866 Yuhangtang Road, Hangzhou 310058, People's Republic of China; 2 Centre for Global Health, Zhejiang University, 866 Yuhangtang Road, Hangzhou 310058, People's Republic of China; 3 UCL Institute for Global Health, 30 Guilford Street, London WC1N1EH, UK

**Keywords:** China, sex ratio, depression, aggression

## Abstract

In parts of rural China male-biased sex ratios at birth, combined with out-migration of women, have led to highly male-biased adult sex ratios, resulting in large numbers of men being unable to marry, in a culture where marriage and reproduction are an expectation. The aim of this study was to test the hypotheses that older unmarried men are more predisposed to depression, low self-esteem and aggression than both those who are married, and those who are younger and unmarried. Self-completion questionnaires were administered among men aged 20–40 in 48 villages in rural Guizhou province, southwestern China. Tools used included the Beck Depression Inventory, the Rosenberg's Self-esteem Scale and the Bryant-Smith Aggression Questionnaire. Regression models assessed psychological wellbeing while adjusting for socio-demographic variables. Completed questionnaires were obtained from 957 never-married men, 535 married men aged 30–40, 394 partnered men and 382 unpartnered men aged 20–29. After adjusting for socio-demographic variables, never-married men were more predisposed to depression (*p* < 0.05), aggression (*p* < 0.01), low self-esteem (*p* < 0.05) and suicidal tendencies (*p* < 0.001). All the psychological measures deteriorated with age in never-married men. In contrast, married men remained stable on these dimensions with age. Never-married men are a psychologically highly vulnerable group in a society where marriage is an expectation. Since the highest birth sex–ratio cohorts have not yet reached reproductive age, the social tragedy of these men will last for at least another generation.

This article is part of the themed issue ‘Adult sex ratios and reproductive decisions: a critical re-examination of sex differences in human and animal societies’.

## 1. Background

In this paper we explore the effects of some of the most extreme human adult sex ratios (ASR) in the world today. Across China, a surfeit of males, particularly in rural areas, leads to a lack of marital prospects for large numbers of men. This situation is of contemporary concern, both in the public consciousness and research literature, and has recently been described as a ‘real world crisis of monumental proportions’ [1]. Through our work, we focus on understudied outcomes related to the mental health and aggressive tendencies of unmarried men, in a cultural context where there is a family obligation to marry. Our findings provide insight into the current debate regarding the negative consequences of populations with a surplus of males.

The extremely male-biased ASRs seen in rural China are the result of a combination of male-biased sex ratios at birth (SRB) and out-migration of women. Male-biased SRBs are seen in societies where there is a tradition of son preference, easy access to sex-selective technology and a low fertility culture [2]. Such male-biased sex ratios are present primarily in Asian countries, including South Korea, Taiwan, India and, in particular, China. China is unique in having experienced mandated low fertility for the past 35 years, because of the One Child Policy. This policy restricted urban individuals to one child and the rural individuals to no more than two, depending on local circumstances. Consequently, the One Child Policy, combined with a strong tradition of son preference, led to the most male-biased SRB in the world for at least two decades. Defined as males/(males + females), the sex ratio at birth rose from 0.52 in 1982, peaked at 0.55 in 2005, and declined to 0.54 in 2010. The latest national estimates put the SRB at around 0.53 in 2015 [3]. However, these averages hide wide disparities across the country from normal levels in western regions (less than 0.52), to ratios of up to 0.58 in poor inland provinces in the south and east of the country. Consequently, by 2020 there will be an excess of an estimated 20 million men of reproductive age in China (which equates to approximately 135 million men to 115 million women) [4].

Further compounding the ASR imbalance in poor, rural areas is out-migration of women. For nearly two decades, increasing numbers of young adults from rural areas have moved to urban areas to seek work. Nearly half of these 260 million rural-urban migrants are female. In China, and several other Asian countries, hypergamy is common (i.e. where women may ‘marry-up’ in social class), resulting in a large proportion of female migrants marrying men in urban areas, and thereby leaving their natal villages permanently [5,6]. Because male migrants typically return to their villages of origin, hypergamy further depletes females from rural areas. Thus, in the prevailing marriage market where women are rare, brides and their families can place hefty financial demands for marriage on potential suitors that make it virtually impossible for the poorest men to marry [1]. Consequently, it is largely uneducated, rural and poor male peasants who are left behind.

Although rapid modernization has eroded cultural demands to marry in some larger urban areas, in smaller cities and in rural China, to marry and become a parent is still crucial to an individual's identity, well-being and family acceptance [6]. In rural areas, women generally marry (with exceptions being those women with a chronic illness or disability) and it is a convention for men and women to marry their first sexual partner, who may have been chosen by their parents [7]. Co-habitation before marriage is very rare, and while divorce is on the rise across China, it is still relatively uncommon in rural areas of inland provinces [8]. For men, in rural areas in particular, having a wife and at least one child is essential for their literal and figurative survival. For example, wives and children form the family labour force during middle-age, children then provide support for parents in old age, and sons carry the patriline into the future [6,9]. An additional point is that in most of rural China homosexuality is highly stigmatized, and to ‘come-out’ is virtually unknown. So, with little opportunity for either same- or opposite-sex pairing, a male-biased sex ratio leads de facto to large numbers of unmarried men.

Much of the research on the consequences of unbalanced sex ratios draws on sociological, psychological and evolutionary theory (as outlined in other contributions to this issue). In China the sheer numbers of unmated men have been a cause for concern [10]. It is theorized that an excess of males, with their generally elevated levels of aggression and violence (particularly over partners), will increase social and familial instability in a population [11,12]. There is good reason to expect this. For example, cross-cultural evidence shows that the overwhelming majority of violent crime is perpetrated by young, unmarried, low-status males [13]. The relationship between ASR and violence has been widely studied, including in very male-biased sex ratio countries, such as India [14,15] and China [10,16]. A recent review by Schacht, Rauch and Borgerhoff Mulder on the relationship between the ASR and male violence finds that male-biased ASRs are at best inconsistently associated with elevated rates of crime and violence [17]. However, the studies in this review nearly all drew data from secondary sources, such as census data for the sex ratio, and police reports (often combining different types of crime) for outcome.

Furthermore, based on these assumptions that unpartnered males have a tendency to violence, and because these men may lack a stake in the existing social order, it is feared that they will become bound together in an outcast culture, turning to antisocial behaviour and organized crime [13], thereby threatening societal stability and security [11]. Kanazawa theorized that intergroup conflict and civil war could erupt [18], with Hudson & Den Boer going even further, predicting that such men will be attracted to military-type organizations, potentially triggering large-scale domestic and international violence [11]. These assumptions have acquired considerable credence to the extent that in January 2007, the Central Committee of the State Council in China declared that the sex imbalance ‘will affect social stability’ [19].

While the effects of male-biased sex ratios on violence and crime have been the focus of many studies, the mental health consequences for men who do not marry or have children, especially in traditional societies, are understudied. It is hypothesized that being forced to remain single under these conditions may result in low self-esteem (that is, lack of confidence in one's worth or abilities) and increased susceptibility to psychological stress, including suicidal tendencies [20–27]. Other work on mental health and sex ratios in Japan finds a positive association between male-biased sex ratios and suicide rates [28].

Through this project, we seek to fill a research gap related to the consequences of sex ratio imbalance. Here we define the sex ratio. Much ASR work focuses on populations where the sex ratio is only marginally skewed, such as the USA, [17,29,30] (and where there is a reliance on secondary data sources), whereas few studies explore the effect of highly unbalanced sex ratios of 0.6 and over, with notable exceptions [31]. In our previous qualitative research we observed that after the age of about 30, men in rural areas realized they were unlikely to marry [25,26]. Accordingly, to explore this further, here we focus on men who have no prospect for marriage and the resultant consequences related to mental health and aggression. We predict that older, unmarried men will express depression, low self-esteem and aggression at higher rates than those who are married, as well those who are younger and unmarried, yet still have time to secure a marital partner.

## 2. Methods

The study was part of a large multidisciplinary research programme exploring the impact of high sex ratios on populations and society in China. This part of the study was conducted in September to December 2015 in Guizhou Province in southwest China, where the sex ratio in the 20–40 age group is estimated at 118 overall, although there are estimates as high as 150 in many rural localities [3]. We applied a cross-sectional survey comparing four groups of men defined by marital/partnership status and age. Because our previous research in rural areas with male-biased ASRs showed that unmarried men over the age of 30 are generally regarded as unmarriageable, we defined an un-partnered man over the age of 30 as a ‘never-married man’ (i.e. likely to never marry). In keeping with the de facto reproductive age range in rural China, we limited the age to under 40. For the purposes of comparison, we included three additional groups:

(1) partnered/married men aged 20–29
(2) unpartnered/unmarried men aged 20–29
(3) partnered/married men aged 30 and over
(4) never-married men aged 30 and over.

For simplicity and readability, we use the terms young partnered and young unpartnered for groups 1 and 2, and married and never-married for groups 3 and 4. For our analysis of trends across the age range, the sample was further divided into partnered and non-partnered men, by age: 20–24, 25–29, 30–34, 35 and above.

The study sample was achieved through multistage stratified probability sampling. The major administrative division of rural China is the county, with a median population in Guizhou of around 400 000. Within counties there is a three-tier population structure: the county urban centre (called the county), the township and the village. We randomly selected two counties in Guizhou province with ASRs of at least 0.6. We then randomly selected six townships within each county and four villages in each town, giving a total of 48 villages.

Establishing accurate ASRs of current residents at the village level can be challenging due to the dynamic nature of migration. Fortunately, village leaders keep and update detailed vital and residence statistics for all individuals registered in their village. Thus, we were able to obtain figures for unmarried individuals who were registered as inhabitants in 36 of the 48 villages in which we conducted this research. In table 1 we present ASR by age group to highlight that for men over the age of 30, the relative numbers of available women are very low.

**Table 1.** Absolute numbers of unmarried males and females resident in 36 villages in Guizhou province by age.

age (years)	males	females	ratio unmarried males: females
20–24	1986	1010	2.0
25–29	956	342	2.8
30–34	468	35	13.4
35–39	302	4	75.5

From the lists of inhabitants provided by village leaders we randomly sampled men aged 20–40 years, stratified by marital status. We aimed to investigate roughly 1000 never-married men, and 400–500 in each of the other three groups. The research team spent 3–4 days in each village, and all men in the age range resident at the time were approached at home by a member of the research team. The study was explained to them before written informed consent was obtained. Participants then completed a questionnaire. A researcher was on hand to provide help for semi-literate respondents. Participants were assured of anonymity, confidentiality and the right to refuse participation.

### (a) Measures

#### (i) Socio-demographic

The questionnaire is in the Electronic Supplementary Material. The socio-demographic variables included age, ethnicity, education level and perception of wealth. The majority (92%), of the Chinese population is Han Chinese, but in Guizhou there are many different ethnicities, though each group is relatively small in number, and around 20% of individuals are of mixed ethnicity. Accordingly, we combined all ethnic groups to create a dichotomous variable, Han and non-Han. Because of the non-cash economy in many of the study areas, we asked about perceived relative wealth instead of income as a measure of participants' relative economic status: ‘How do you feel your level of household wealth compares with the average in your community?’

#### (ii) Depression

Depression was measured using the Chinese version of the Beck Depression Inventory (BDI), which has been widely used and validated in Chinese populations [32–35]. It consists of 21 items, including feelings of sadness, guilt, suicidal thoughts and wishes, and insomnia. Respondents were asked to score their feelings over the previous month on a four-point Likert scale from ‘0’ meaning not having that feeling or symptom at all, to ‘3’, having it very strongly. The total score ranges from 0 to 63, with higher scores indicating greater depression. The BDI can be analysed as a categorical and a continuous variable. The categorical variable specifies four levels of depressive tendencies: 0–10, none; 11–20 points, mild; 21–30 points, moderate; and 31–63 points, severe. We use this to estimate the numbers of individuals at risk. As a continuous variable, the mean is a measure of depressive tendency or subjective well-being in a population [35]. We analysed as continuous and categorical variables because of the differing interpretation, that is, the categorical variable estimates the proportion of individuals experiencing depression, while the continuous variable provides an estimate of depression tendency in the population, which can then be compared with other populations. For this study we also separately analysed the question on suicidal thoughts and wishes because of the importance of this item. The Cronbach *α* of the scale was 0.90 for the total sample (*n* = 2268).

#### (iii) Self-esteem

Self-esteem was measured with the Chinese version of the Rosenberg's Self-esteem Scale which has been widely validated in Chinese populations [36]. It consists of 10 items exploring aspects of self-esteem including self-confidence and self-liking. It contains five positively worded items (e.g. ‘I feel that I have a number of good qualities’) and five negatively worded items (e.g. ‘At times I feel that I am no good at all’). Responses range from ‘1’ meaning strongly disagree to ‘4’ meaning strongly agree with positively worded item reverse coded. The score ranges from 10 to 40 with higher scores indicating a higher level of self-esteem. The mean score indicates overall self-esteem in a population, with scores below 25 denoting a tendency to low self-esteem [36]. Again because of the differing use of the continuous and categorical variables we analysed both. The Cronbach *α* of the scale was 0.70 for our sample.

#### (iv) Aggression

Aggression was assessed with the shortened version of the Buss–Perry Aggression Questionnaire (AQ) developed by Bryant and Smith [37]. This shortened version has been used in a number of countries, and has been found to be psychometrically superior to the longer version [38]. It is a 12-item scale that measures four aspects of aggression: anger, hostility, physical aggression and verbal aggression. Aggression is regarded as the behavioural manifestation of anger and hostility, and involves hurting or harming others, either physically or verbally. Anger involves physiological arousal and preparation for aggression, and represents the emotional component of behaviour. Hostility refers to negative attitudes, and represents the cognitive component of behaviour [37]. Each AQ item was rated on a 5-point scale from 1 (extremely uncharacteristic of me) to 5 (extremely characteristic of me) Responses were summed to generate subscale scores (each ranging from 3–15) and a total scale score (ranging from 12–60). The AQ is treated as a continuous variable. A Chinese version is available and has shown good construct validity and internal reliability [38]. The Cronbach *α* for our sample was 0.71.

### (b) Data analysis

Self-esteem and depression were analysed as continuous and categorical variables and aggression and suicide as categorical only. Chi-square tests and one-way ANOVA were used to compare the four groups. Multiple regression models were generated to examine the effects of marital/partnership status on self-esteem, depression, suicidal thoughts and aggressive tendency as dependent variables, while controlling for ethnicity, education level and perceived wealth. For our analysis of trends across the age range, the total sample was divided into partnered and non-partnered men, and four age groups: 20–24, 25–29, 30–34, 35 and above. All data were analysed with SPSS v. 20.0.

## 3. Results

### (a) Socio-demographic characteristics of the sample

Completed questionnaires were obtained from 2268 participants: 957 (42.2%) never-married aged 30–40, 535 (23.6%) married aged 30–40, 382 (16.8%) non-partnered aged 20–29 and 394 (17.4%) partnered aged 20–29. Table 2 shows their socio-demographic characteristics. Of the total, 59.9% were Han Chinese, with the remainder from other ethnic groups, of which the most numerous were: the Miao 335 (14.8%), Buyi 204 (9.0%) and Dong 122 (5.4%). There were no significant differences in marital status by ethnicity. Never-married men had much lower levels of education: 57.8% had only primary school education compared with 20.7% in married men (30–40). In the younger cohort this figure was 11.7% in partnered men (20–29), and 7.4% in non-partnered men (*p* < 0.001). Never-married men also had much lower self-reported wealth: 46.4% poorer/much poorer compared with 18.8% in married men, 19.8% in young partnered men and 32.9% in young non-partnered men.

**Table 2.** The socio-demographic characteristics of the participants.

	never-married men (30–40) *n* = 957	married men (30–40) *n* = 535	unpartnered men (20–29) *n* = 382	partnered men (20–29) *n* = 394	*F* or *χ^2^*	*p*
age in years (*mean* *±* *s.d.*)	34.2 ± 2.9	34.9 ± 3.0	23.6 ± 2.1	24.7 ± 2.5		
nationality *%*(*n*)					5.796	0.122
Han	60.0 (574)	55.1 (292)	62.6 (236)	58.8 (228)		
other minorities	40.0 (382)	44.9 (238)	37.4 (141)	41.2 (160)		
education *%*(*n*)					829.4	0.000
illiteracy and primary school	57.8 (552)	20.7 (107)	7.4 (28)	11.7 (45)		
middle school	26.6 (254)	55.2 (285)	30.1 (114)	38.7 (149)		
high school	9.5 (91)	17.8 (92)	11.1 (42)	14.3 (55)		
junior college and above	6.1 (58)	6.2 (32)	51.5 (195)	35.3 (136)		
income level *%*(*n*)					150.6	0.000
much better off/better-off/the same	53.6 (512)	81.2 (397)	67.1 (245)	80.2 (296)		
poorer/much poorer	46.4 (444)	18.8 (92)	32.9 (120)	19.8 (73)		

### (b) Self-esteem, depression and aggression by age and marital status

Table 3 compares the self-esteem, depression, aggression and suicidal thoughts/wishes of never-married men with the other three groups. The mean depression scores in never-married men (17.2) were significantly higher than other three groups: 12.3 in married men, 12.6 in young non-partnered men and 12.7 in young partnered men. Self-esteem scores in never-married (25.9) were significantly lower than married (27.2), young partnered (27.7) and young non-partnered (27.6). The total aggression score (36.4), and all four subcategories of aggression (8.4 in physical aggression, 9.1 in verbal aggression, 9.4 in anger and 9.6 in hostility) were also significantly higher in never-married men. The self-esteem and depression scores were also analysed as categorical variables in order to identify the proportions of vulnerable individuals. The proportion of low self-esteem among never-married men (32.5%) was over twice that in the other three groups (15.5% in married, 14.4% in young partnered and 15.0% in young non-partnered). The proportion of never-married men with moderate-severe depression was 38.5%, also almost twice as high as in the other three groups (21.5% in married, 21% in young partnered and 19.9% in young non-partnered). In terms of suicide ideation 11% of never-married men said they would like to kill themselves or would if they had the chance. This is around three times the rate for the other three population groups (3.2% in married, 3.8% in young partnered and 3.8% in young non-partnered).

**Table 3.** Self-esteem scores (SES), Beck Depression Inventory Scores (BDI) and Aggression Scores (AQ): comparison of four groups.

	never-married men (30–40)	married men (30–40)	unpartnered men (20–29)	partnered men (20–29)	*F* or *χ^2^*	*p*
SES (mean *±* s.d.)	25.9 ± 3.3	27.2 ± 3.3	27.7 ± 3.5	27.6 ± 3.5	42.05	0.000
SES (categorical)*%(n)*					96.31	0.000
low (<25)	32.5 (311)	15.5 (79)	15.0 (56)	14.4 (55)		
high (≥25)	67.5 (645)	84.5 (432)	85.0 (318)	85.6 (328)		
BDI (mean *±* s.d.)	17.2 ± 11.3	12.3 ± 10.6	12.6 ± 10.1	12.9 ± 10.6	33.48	0.000
BDI (categorical)*%(n)*					108.8	0.000
low risk for depression (≤10)	32.4 (310)	52.7 (272)	50.9 (192)	49.9 (195)		
mild depression (11–20)	29.1 (278)	25.8 (133)	29.2 (110)	29.2 (114)		
moderate depression (21–30)	24.5 (234)	13.6 (70)	13.8 (52)	12.3 (48)		
severe depression (>30)	14.0 (134)	7.9 (41)	6.1 (23)	8.7 (34)		
AQ (mean *±* s.d.)	36.4 ± 6.8	33.5 ± 7.9	33.7 ± 7.6	34.3 ± 7.8	23.87	0.000
physical aggression (mean *±* s.d.)	8.4 ± 2.5	7.1 ± 2.8	7.4 ± 2.7	7.7 ± 2.7	30.98	0.000
verbal aggression (mean *±* s.d.)	9.1 ± 2.2	8.7 ± 2.5	8.5 ± 2.2	8.7 ± 2.4	8.000	0.000
anger (mean *±* s.d.)	9.4 ± 2.5	8.6 ± 2.7	8.4 ± 3.0	8.8 ± 2.8	16.59	0.000
hostility (mean *±* s.d.)	9.6 ± 2.4	9.1 ± 2.7	9.4 ± 2.5	9.1 ± 2.6	5.195	0.001
suicidal thoughts and wishes *%(n)*					104.2	0.000
I don't have any thoughts of killing myself	62.5 (596)	82.4 (416)	80.3 (302)	77.3 (300)		
I have thoughts of killing myself, but I would not carry them out	26.5 (253)	14.5 (73)	16.0 (60)	18.8 (73)		
I would like to kill myself	6.6 (63)	1.0 (5)	1.9 (7)	1.5 (6)		
I would kill myself if I had the chance	4.4 (42)	2.2 (11)	1.9 (7)	2.3 (9)		

Regression models showed that never-married men were significantly more likely to have depression (*p* < 0.05), aggressive tendencies cumulatively, and across all four subcategories (*p* < 0.01), low self-esteem (*p* < 0.05) and suicidal tendencies (*p* < 0.001) than all other groups while adjusting for ethnicity, education and perceived comparative wealth (tables 4 and 5).

**Table 4.** Self-esteem, depression and suicidal thoughts: socio-demographic associations. SES: (low self-esteem, *y* = 1; high self-esteem, *y* = 0). BDI: (having mild, moderate and severe depression, *y* = 1; having no depression, *y* = 0). Suicidal thoughts: (I have thoughts of killing myself, but I would not carry them out/ I would like to kill myself/I would kill myself if I had the chance, *y* = 1; I don't have any thoughts of killing myself, *y* = 0).

	SES (continuous)	BDI (continuous)	suicidal thoughts (categorical)
group			
never-married men (Ref)	1	1	1
married men (30–40)	0.67 (0.28, 1.07)**	−4.47 (−5.76, −3.18)***	0.35 (0.26, 0.47)***
non-partnered men (20–29)	0.51 (−0.04, 0.97)*	−2.43 (−3.93, −0.92)**	0.43 (0.30, 0.60)***
partnered/married men (20–29)	0.65 (0.20, 1.10)**	−2.21 (−3.66, −0.76)**	0.54 (0.40, 0.75)***
ethnic groups			
Han (Ref)	1	1	1
other nationality	0.25 (−0.04, 0.54)	−0.08 (−1.76, 0.12)	1.01 (0.85, 1.28)
education			
Illiteracy and primary school (Ref)	1	1	1
middle school	0.75 (0.38, 1.12)***	−0.40 (−1.61, 0.82)	1.01 (0.78, 1.31)
high school	1.23 (0.74, 1.71)***	−1.82 (−3.40, −0.24)	1.03 (0.73, 1.44)
junior college and higher	2.50 (2.01, 3.00)***	−4.89 (−6.47, −3.31)***	0.78 (0.55, 1.10)
income level			
poorer/much poorer (Ref)	1	1	1
much better/better/the same	0.86 (0.55, 1.17)***	−2.02 (−3.02, −1.02)***	0.80 (0.64, 0.98)

*p < 0.05, **p < 0.01, ***p < 0.001.

**Table 5.** Self-esteem, depression, aggression and suicidal thoughts: socio-demographic associations (continued).

	aggression score	physical aggression	verbal aggression	anger	hostility
never-married men (Ref)	1	1	1	1	1
married men (30–40)	−2.66 (−3.54, −1.77)***	−1.29 (−1.61, −0.98)***	−0.45 (−0.73, −0.18)**	−0.61 (−0.94, −0.29)***	−0.30 (−0.60, −0.004)*
unpartnered men (20–29)	−1.92 (−2.96, −0.88)***	−0.66 (−1.03, −0.29)***	−0.35 (−0.67, −0.03)*	−0.80 (−1.18, −0.42)***	−0.11 (−0.46, 0.25)
partnered men (20–29)	−1.20 (−2.20, −0.20)*	−0.32 (−0.67, 0.04)	−0.25 (−0.56, 0.06)	−0.42 (−0.78, −0.05)*	−0.21 (−0.55, 0.13)
*ethnic groups*					
Han (Ref)	1	1	1	1	1
other nationality	−0.54 (−1.19, 0.11)	−0.29 (−0.52, −0.06)*	0.02 (−0.18, 0.22)	0.01 (−0.23, 0.25)	−0.28 (−0.50, −0.06)*
*education*					
illiteracy/primary (ref)	1	1	1	1	1
middle school	−0.50 (−1.33, 0.34)	−0.15 (−0.45, 0.15)	0.07 (−0.19, 0.33)	−0.23 (−0.54, 0.07)	−0.18 (−0.47, 0.10)
high school	0.18 (−0.92, 1.27)	−0.13 (−0.52–0.26)	0.26 (−0.08, 0.60)	0.02 (−0.37, 0.42)	0.02 (−0.35, 0.39)
junior college and higher	−1.75 (−2.84, −0.66)**	−0.63 (−1.02, −0.25)**	−0.51 (−0.85, −0.17)**	−0.51 (−0.91, −0.12)*	−0.09 (−0.46, 0.28)
*income*					
poorer (ref)	1	1	1	1	1
much better/better/the same	−0.42 (−1.11, 0.28)	−0.01 (−0.26, 0.23)	0.03 (−0.18, 0.25)	−0.10 (−0.35, 0.16)	−0.34 (−0.57, −0.10)**

*p < 0.05, **p < 0.01, ***p < 0.001.

**Figure 1.**
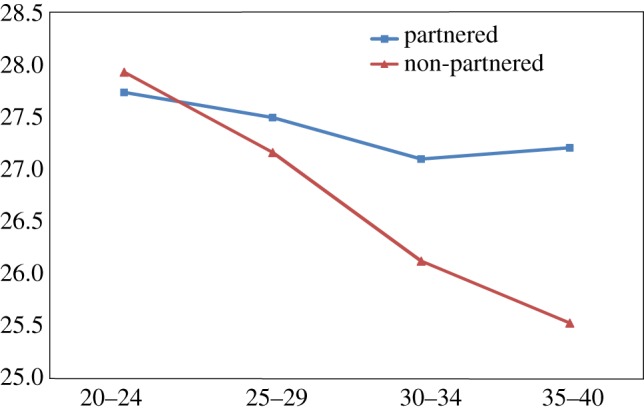
Self-esteem score (SES) of partnered and non-partnered men by age (*Y* axis, mean of SES score). (Online version in colour.)

**Figure 2.**
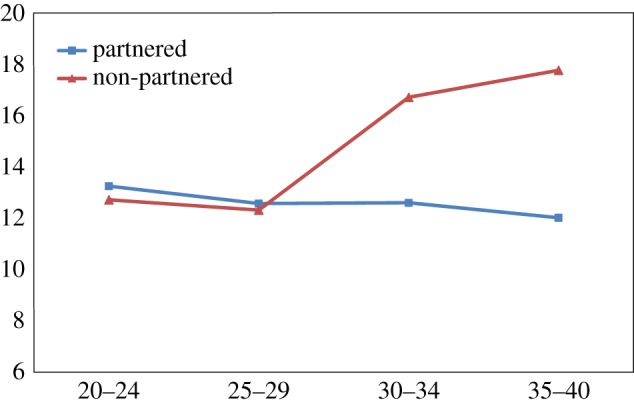
Scores for the Beck Depression Inventory of partnered and non-partnered men by age (*Y* axis, mean of BDI score). (Online version in colour.)

**Figure 3.**
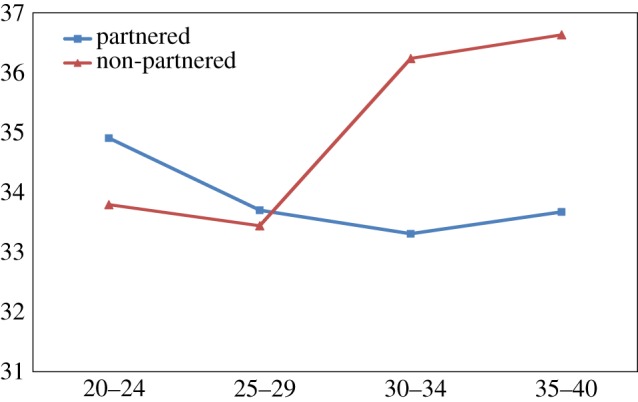
Aggression scores of partnered and non-partnered men by age. (*Y* axis, mean of AQ score). (Online version in colour.)

Finally, there was a steady gradient of reduced self-esteem, increased depression and increased aggression among non-partnered men with increasing age, across all four age groups, 20–24, 25–29, 30–34 and 35–40. In contrast married men remained little changed on these dimensions with age (figures 1–3).

## 4. Discussion

The very male-biased ASRs in this area of China, all well in excess of 0.6, are among the highest in the world today, and comparable to those seen in early settler populations [39]. Clearly, the permanent out-migration of women contributes hugely to an already distorted sex ratio caused largely by prenatal sex-selective abortion. Our study sheds light on the reality of these men's lives. These unmarriageable men represent the victims of a distortion of the sex ratio driven by a perfect storm of socio-cultural and economic factors. We show that large numbers of surplus men who will be unable to marry are more vulnerable to depression, with a greater tendency to suicidal ideation. They have lower self-esteem and are more likely to express aggressive tendencies. Among the four groups the never-married men consistently stand out as much more psychologically vulnerable. Our findings highlight a number of important issues, and generate predictions about the behavioural response to unbalanced sex ratios.

Firstly, we show that in male-biased sex ratio settings, opportunities for marriage are diminished for men of low socio-economic status. This phenomenon was observed in Pollett's historical study from the USA in the early twentieth century [40], where local high sex ratios meant that women were able to ‘drive a hard bargain’ in their choice of mate, with men of low socio-economic status unable to compete. In China this is magnified by the expectation that men will provide for the new bride, a de facto bride price [7]. Where possible, the parents of these men focus on saving to improve their sons' stakes in the mating market. This phenomenon of saving for marriage of sons has been shown to have increased with the rise in the sex ratio in China [41,42]. In conducting this research we learnt that in families with two sons, parents would usually make efforts to ensure that at least one was able to marry. This often involved unfair distribution of land and economic destitution for the unfavoured son, a situation described by Huang in Hebei province [43].

Given that low socio-economic status is an established correlate for depression and low self-esteem [44,45] and for difficulty in attracting a partner, these relationships are complex and intertwined. This also raises the question about direction of causation. It seems intuitive that men who are depressed, aggressive and lacking in confidence, are more likely to have difficulty attracting a partner, especially in a situation where women are scarce. But while our findings are correlative, we show that young partnered and non-partnered men score similarly on psychological testing and education level, though young partnered men score slightly higher for perceived wealth. We believe this mainly reflects the homogeneity of this rural population. Many of these young men may start out believing that they will have an opportunity in the mating market, despite the shortage of females in these villages. But then, as already mentioned, many young rural inhabitants, male and female, migrate to cities to work, males usually on a temporary basis. Those men who stay behind are more likely to have land and to marry local women who do not migrate. Young unpartnered men who leave their villages for study and work increase their ‘value’ in a competitive marriage market through education and accruing savings, and hence remain optimistic about their chances [41].

Older men who had never-married had strikingly lower levels of education than married older men. This relates to a very strong cohort effect for education. Access to secondary education improved hugely in these areas during the 1990s. Older men who had obtained a secondary education when it was less accessible had a clear advantage in the mating market over those who had just primary education.

Secondly, while the literature from a range of cultures shows that married individuals have higher levels of well-being than unmarried, with these effects more marked in men [46,47], our study shows that the negative effects of not being married or in a long-term partnership are higher than reported in other settings, including the USA and Japan [48]. Research from these countries showed depression scores were only marginally higher in unmarried men, explained in part by the fact that the unmarried state is much more socially acceptable in both settings. In contrast, rural China remains staunchly traditional, and not marrying is viewed as a personal failure. This is compounded by pressures from parents and relatives to get married, lack of shared interests with peers whose focus is the family, leading to narrowing of social networks and isolation. These phenomena may progressively erode self-esteem, while leading to depression and tendency toward aggression [49]. Our study shows that these effects become more marked after the age of 30, when marriage becomes unlikely in this population.

Thirdly, our findings throw light on the controversy around the association between high ASRs and violence [17]. It should be noted that the aggression scores across all four groups are around double those in male populations elsewhere [50,51]. For example, Norwegian males scored a total of 16.7 with 3.6 for physical aggression [50]. Our scores for never-married men are close to Norwegian and US males who have a history of violence [51]. The reasons for this are unclear, but one possible explanation is that the unmarried men in our sample internalize their aggressive tendencies, which leads in turn to stronger feelings of aggression. It has been proposed that this relationship between internalizing symptoms and aggression is mediated by the emotion of anger [52].

This raises questions around the hypothesized association between high sex ratios, aggression and violence. In these villages, levels of societal violence are very low. We also show that younger men, who in most societies are the most likely to commit acts of violence [11], appear to have lower aggressive tendencies than older men. In the context of the high sex ratio, this may be the result of a conscious or subconscious awareness that being aggressive or committing violence may reduce chances in the mating market, especially in those younger men who remain optimistic about mating in the future. The higher levels of aggression (and consistency across all four subscores), which increase with age in the never-married men, we attribute to loss of hope, discrimination and marginalization, family pressure, sexual frustration and loneliness. As noted above, we further hypothesize that this aggression is largely internalized and may help to explain the higher levels of depression, low self-esteem and aggression scores. So despite the high aggression scores, we find no evidence to support the Hudson & Den Boer hypothesis that these unmarried men present a threat to societal stability [11].

Fourthly, the problem associated with excess males in these areas will continue for the next two decades at least. While the highest SRB cohorts have yet to reach reproductive age, son preference even in rural China is declining, and this is already reflected in the decrease in the SRB. While the introduction of the Universal Two Child Policy in January 2016 will have little direct effect in these poor rural areas, since two children are already permitted, the more relaxed approach will probably allow three in such areas, thus reducing further the pressure to sex-select [53]. The change in policy may also help to reduce the sex ratio imbalance in urban areas, thereby reducing the permanent out-migration of women. This is very important because it is the out-migration of women for marriage that makes the major contribution to the imbalanced ASR in these poor rural areas. To restrict rural-urban migration and limit female marriage migration are simply not feasible, given their massive contribution to economic development and the recognized right of individuals to migrate. On the positive side, migration alleviates the, albeit smaller, problem of surplus men in urban areas. But this unmarried underclass of men in poor rural areas will be a social problem for the foreseeable future.

Finally, our findings raise serious concerns about the mental well-being of these men, in a setting where mental health services are virtually non-existent. The finding that 6.6% of the never-married men ‘would like to’ kill themselves and 4.4% would ‘if they could’ is a very serious concern. Currently, where mental health services exist, they focus on severe mental illness, such as psychosis. Moreover, doctors at township and village level are not trained to identify and treat mental health problems. Training rural doctors in common mental health conditions, for example, using psychological screening tools to identify severe depression, would be relatively straightforward. This of course necessitates a referral system, requiring a strengthening of mental health services at higher levels, for example, at county hospitals. Such service provision, which would have benefits well beyond meeting the psychiatric needs of the most vulnerable unmarried men, is long overdue and should be incorporated into the ongoing rural health reforms. Local governments are very aware of the problem of bachelor villages and there is sympathy for the plight of these men. This has already led to guarantees of basic standard of living for never-married men, just as couples with no children receive extra support.

## 5. Limitations

Clearly, the results from two counties from one poor province in China must be extrapolated with caution. The usual caveats about self-report of mental health measures need to be noted. However, members of our research team noted that all participants were conscientious in the completion of the questionnaire and frequently asked for asked for help when items were not understood. While selection bias was a possibility, the very small number of refusals (around 20 of all men approached, across all villages) means the effects of this are minimal. Misclassification of partnering in the younger age groups is possible because of ambiguity about the level of seriousness of a relationship. Having a relationship with someone where there is no formal commitment may or may not have counted as a partner. But again the numbers involved are likely to be small. Finally, we did not include men over the age of about 40, so we cannot comment on whether psychological well-being continues to decline with age, or whether there is growing acceptance of circumstances along with reduced sexual need, and hence improved psychological well-being. More research should elucidate this further.

## Supplementary Material

Questionnaire

## Data accessibility

The data are deposited in the Economic and Social Research Council Research Outcomes now located at Researchfish, www.researchfish.net (grant no. ES/FO34466/1).
