# Supplementary material for: High sex ratios in rural China: declining well-being with age in never-married men
Source: Philos Trans R Soc Lond B Biol Sci. 2017 Jul 31;372(1729):20160324. doi: 10.1098/rstb.2016.0324 (PMC5540864; doi:10.1098/rstb.2016.0324)
Supplement: Questionnaire [file rstb20160324supp1.doc]

**Questionnaire translation (from Chinese)**

1. Sex: ______

2. Nationality: ______

3. Date of Birth: ________Year _____Month

4. Your Current Place of Residence: _______Province _______County（City/District）_________Township（Street）_________Village（[residents committee](javascript:showjdsw('jd_t','j_'))）

5. Your Educational level: Circle the level of education which you have completed

-Not attended school

-Primary School

-Middle School

-High School

-Junior College

-Undergraduate or higher

6. How do you feel your family compares in terms of income with average families in your community?

-much better off

-better off

-the same

-poorer

-much poorer

7. How many siblings do you have altogether? _______

8. How many older sisters____; younger sisters____; older brothers____; younger brothers____

9. Do you have a partner? Y/N

10. If you do not have a partner why do you think this is? (Mark as many as you think apply).

-I’m poor

-I’m very shy

-My home is very remote, and no one would like to live there

-Very bad temperament

-I has a chronic disease or disability

-I has no ambition

-I’m unattractive

-I doesn’t want a partner

-Write down any other reason we haven’t included here

11. How does the community look on you because you still have not found a partner?

(Mark as many as you think apply).

-They think it is my choice

-They think I am not of good character

-They feel sorry for me.

-They think I has bad habits, lazy

-They think I am very fussy.

-They are worried about me and urge me to find a partner.

-No view

12. what have happened to you because you haven’t found a partner?

(Mark as many as you think apply.

-become more introvert

-get more depressed

-keep myself to myself and not mix very much

-be bad-tempered and get angry easily

-feel envious

-drink too much

-enjoy the freedom and lack of responsibility

-be very successful in my work

-go to CSWs

-just the same as if I had a partner.

13. How do local residents view you because you have not been married and had no children? (Mark as many as you think apply)

-other people will feel no different

-my family is prone to be teased

-They will look down on my family

- my parents or relatives would treat me coldly

- Other -say what you think

14. Do you think there are more young men or more young women in China?

- many more young men

-somewhat more young men

-the same/don’t know

-somewhat more young women

-many more young women

15. Do you think there are more men or more women in your local area?

-More men

-more women

-the same

-I don’t know

16. There are a lot of ideas about the effects on society if there are more young men than women. What do you think? Which of the following do you agree with?

1. There will be more street crime

Strongly agree/ Agree / neither agree not disagree/disagree/strongly disagree

1. There will be more sex crime

Strongly agree/ Agree / neither agree not disagree/disagree/strongly disagree

1. There will be more prostitution

Strongly agree/ Agree / neither agree not disagree/disagree/strongly disagree

1. Women will be more valued in society

Strongly agree/ Agree / neither agree not disagree/disagree/strongly disagree

1. Women will be more valued in their families

Strongly agree/ Agree / neither agree not disagree/disagree/strongly disagree

1. There will be more extramarital sex

Strongly agree/ Agree / neither agree not disagree/disagree/strongly disagree

1. There will be more premarital sex

Strongly agree/ Agree / neither agree not disagree/disagree/strongly disagree

1. There will be more homosexuality

Strongly agree/ Agree / neither agree not disagree/disagree/strongly disagree

1. There will be more trafficking of women

Strongly agree/ Agree / neither agree not disagree/disagree/strongly disagree

**Self-esteem:**

1. On the whole, I am satisfied with myself.

Strongly agree/ Agree / neither agree not disagree/disagree/strongly disagree

1. At times, I think I am no good at all.

Strongly agree/ Agree / neither agree not disagree/disagree/strongly disagree

1. I feel that I have a number of good qualities.

Strongly agree/ Agree / neither agree not disagree/disagree/strongly disagree

1. I am able to do things as well as most other people.

Strongly agree/ Agree / neither agree not disagree/disagree/strongly disagree

1. I feel I do not have much to be proud of.

Strongly agree/ Agree / neither agree not disagree/disagree/strongly disagree

1. I certainly feel useless at times.

Strongly agree/ Agree / neither agree not disagree/disagree/strongly disagree

1. I feel that I’m a person of worth, at least on an equal plane with others.

Strongly agree/ Agree / neither agree not disagree/disagree/strongly disagree

1. I wish I could have more respect for myself.

Strongly agree/ Agree / neither agree not disagree/disagree/strongly disagree

1. All in all, I am inclined to feel that I am a failure.

Strongly agree/ Agree / neither agree not disagree/disagree/strongly disagree

1. I take a positive attitude toward myself.

Strongly agree/ Agree / neither agree not disagree/disagree/strongly disagree

**Depression**

1.

0 I do not feel sad.

1 I feel sad

2 I am sad all the time and I can't snap out of it.

3 I am so sad and unhappy that I can't stand it.

2.

0 I am not particularly discouraged about the future.

1 I feel discouraged about the future.

2 I feel I have nothing to look forward to.

3 I feel the future is hopeless and that things cannot improve.

3.

0 I do not feel like a failure.

1 I feel I have failed more than the average person.

2 As I look back on my life, all I can see is a lot of failures.

3 I feel I am a complete failure as a person.

4.

0 I get as much satisfaction out of things as I used to.

1 I don't enjoy things the way I used to.

2 I don't get real satisfaction out of anything anymore.

3 I am dissatisfied or bored with everything.

5.

0 I don't feel particularly guilty

1 I feel guilty a good part of the time.

2 I feel quite guilty most of the time.

3 I feel guilty all of the time.

6.

0 I don't feel I am being punished.

1 I feel I may be punished.

2 I expect to be punished.

3 I feel I am being punished.

7.

0 I don't feel disappointed in myself.

1 I am disappointed in myself.

2 I am disgusted with myself.

3 I hate myself.

8.

0 I don't feel I am any worse than anybody else.

1 I am critical of myself for my weaknesses or mistakes.

2 I blame myself all the time for my faults.

3 I blame myself for everything bad that happens.

9.

0 I don't have any thoughts of killing myself.

1 I have thoughts of killing myself, but I would not carry them out.

2 I would like to kill myself.

3 I would kill myself if I had the chance.

10.

0 I don't cry any more than usual.

1 I cry more now than I used to.

2 I cry all the time now.

3 I used to be able to cry, but now I can't cry even though I want to.

11.

0 I am no more irritated by things than I ever was.

1 I am slightly more irritated now than usual.

2 I am quite annoyed or irritated a good deal of the time.

3 I feel irritated all the time.

12.

0 I have not lost interest in other people.

1 I am less interested in other people than I used to be.

2 I have lost most of my interest in other people.

3 I have lost all of my interest in other people.

13.

0 I make decisions about as well as I ever could.

1 I put off making decisions more than I used to.

2 I have greater difficulty in making decisions more than I used to.

3 I can't make decisions at all anymore.

14.

0 I don't feel that I look any worse than I used to.

1 I am worried that I am looking old or unattractive.

2 I feel there are permanent changes in my appearance that make me look unattractive

3 I believe that I look ugly.

15.

0 I can work about as well as before.

1 It takes an extra effort to get started at doing something.

2 I have to push myself very hard to do anything.

3 I can't do any work at all.

16.

0 I can sleep as well as usual.

1 I don't sleep as well as I used to.

2 I wake up 1-2 hours earlier than usual and find it hard to get back to sleep.

3 I wake up several hours earlier than I used to and cannot get back to sleep.

17.

0 I don't get more tired than usual.

1 I get tired more easily than I used to.

2 I get tired from doing almost anything.

3 I am too tired to do anything.

18.

0 My appetite is no worse than usual.

1 My appetite is not as good as it used to be.

2 My appetite is much worse now.

3 I have no appetite at all anymore.

19.

0 I haven't lost much weight, if any, lately.

1 I have lost more than five pounds.

2 I have lost more than ten pounds.

3 I have lost more than fifteen pounds.

20.

0 I am no more worried about my health than usual.

1 I am worried about physical problems like aches, pains, upset stomach, or constipation.

2 I am very worried about physical problems and it's hard to think of much else.

3 I am so worried about my physical problems that I cannot think of anything else.

21.

0 I have not noticed any recent change in my interest in sex.

1 I am less interested in sex than I used to be.

2 I have almost no interest in sex.

3 I have lost interest in sex completely.

**Aggression**

1. I can’t help getting into arguments when people disagree with me.

Strongly agree/ Agree / neither agree not disagree/disagree/strongly disagree

1. I wonder why sometimes I feel so bitter about things.

Strongly agree/ Agree / neither agree not disagree/disagree/strongly disagree

1. I have threatened people I know.

Strongly agree/ Agree / neither agree not disagree/disagree/strongly disagree

1. I flare up quickly but get over it quickly.

Strongly agree/ Agree / neither agree not disagree/disagree/strongly disagree

1. Given enough provocation, I may hit another person.

Strongly agree/ Agree / neither agree not disagree/disagree/strongly disagree

1. At times I feel I have gotten a raw deal out of life.

Strongly agree/ Agree / neither agree not disagree/disagree/strongly disagree

1. I have trouble controlling my temper.

Strongly agree/ Agree / neither agree not disagree/disagree/strongly disagree

1. I often find myself disagreeing with people.

Strongly agree/ Agree / neither agree not disagree/disagree/strongly disagree

1. Other people always seem to get the breaks.

Strongly agree/ Agree / neither agree not disagree/disagree/strongly disagree

1. There are people who pushed me so far that we came to blows.

Strongly agree/ Agree / neither agree not disagree/disagree/strongly disagree

1. My friends say that I’m somewhat argumentative.

Strongly agree/ Agree / neither agree not disagree/disagree/strongly disagree

1. Sometimes I fly off the handle for no good reason.

Strongly agree/ Agree / neither agree not disagree/disagree/strongly disagree
